# Bioelectrical impedance analysis of body composition in children and adolescents with type 1 diabetes: a prospective case–control study

**DOI:** 10.1007/s00431-025-06401-4

**Published:** 2025-08-18

**Authors:** S. Giardinelli, A. G. Lambertini, A. Lumaca, D. Boschiero, F. Cavallin, S. Zucchini, C. Malaventura, A. Suppiej

**Affiliations:** 1 School of Pediatrics, Department of Medical Sciences, University of Ferrara, Ferrara, Italy; 2 Diabetes and Endocrinology Clinic, Pediatric Unit, University Hospital, Arcispedale Sant’Anna , Ferrara, Italy; 3 BioTekna®, Marcon-Venice, 30020 Italy; 4 Independent Statistician, Solagna, 36020 Italy; 5 Pediatric Section, Department of Medical Sciences, University of Ferrara, Ferrara, Italy; 6 Pediatric Unit, University Hospital, Arcispedale Sant’Anna, Ferrara, Italy

**Keywords:** Type 1 diabetes, Body composition, BIA-ACC, Phase angle, Physical activity, Children

## Abstract

**Abstract:**

Children and adolescents with type 1 diabetes can have impaired body composition. The aim of this study was to assess and compare body composition and phase angle, a cellular health indicator, in young patients with diabetes and healthy peers using a dual-frequency bioelectrical impedance analysis. Moreover, the influence of physical activity, glycemic control, disease duration, insulin dose, and delivery mode on body composition were explored. This was a prospective case–control study conducted on 46 patients with diabetes and 92 healthy subjects matched on the basis of pubertal status. BIA-ACC® analyzer, a dual-frequency device, was used to perform bioelectrical impedance analysis, and fat mass index and percentages of fat mass, free fat mass, total water content, extracellular and intracellular water, as well as basal metabolic rate and phase angle, were assessed; in patients with diabetes, information about disease duration, glycated hemoglobin, time in range, time above range, time below range, total daily insulin dose, and insulin delivery mode were collected. Patients with diabetes showed lower phase angle than controls, and the median phase angle of patients with diabetes that practiced extracurricular physical activity was comparable to healthy subjects. No other body composition parameters differed between cases and controls. Longer disease duration and higher daily insulin dose were both correlated with higher basal metabolic rate and higher phase angle.

**Conclusion:**

Children and adolescents with good management of type 1 diabetes showed comparable body composition measurements but a lower phase angle to their healthy peers. Patients with diabetes practicing extracurricular physical activity had phase angle values comparable to healthy subjects.

**Supplementary Information:**

The online version contains supplementary material available at 10.1007/s00431-025-06401-4.

## Introduction

Assessing and maintaining optimal body composition is of particular importance in people suffering from type 1 diabetes (T1D), as higher fat and lower muscular masses have been described in patients with diabetes [1–3].

Bioelectrical impedance analysis (BIA) is a widely used method to estimate body composition, as it is non-invasive, safe, cost-effective, and rapid, and can be performed by operators after minimal training [4]. Its functioning is based on different conductivity, permittivity, and resistivity properties of each body compartment, estimated by applying alternating low-voltage electrical current to the body [5]. Advanced BIA devices can precisely estimate muscle, bone, extracellular tissue, and energy balance [6]. BIA has been previously used in both healthy and ill children to assess the impact of various diseases on body composition, hydration status, and also on cellular health, through the assessment of phase angle (PA°) [7]. PA° represents the relationship between resistance and reactance in body tissues and provides information about cellular performance and body water distribution between extracellular and intracellular spaces, with low values suggesting altered integrity and permeability of cell membranes [8].

The literature offers few studies assessing body composition with BIA in pediatric patients with diabetes, which have been performed at diabetes onset [9] or in patients not using diabetes technology [10]. Consequently, such investigations should be extended to patients with different disease durations and using advanced technologies to monitor the disease, such as continuous glucose monitoring (CGM) or flash glucose monitoring (FGM), and pumps to deliver insulin.

This study employed a dual-frequency bioimpedance device (BIA-ACC®) to accurately assess several parameters of body composition in T1D children and adolescents compared to a group of healthy peers. In addition, we explored the influence of physical activity, glycemic control, disease duration, insulin dose, and type of insulin therapy on body composition.

## Methods

### Study Design and Participants

This was a prospective, case–control study. Cases were children and adolescents with T1D referred to the Pediatric Endocrinology and Diabetes Clinic of the Pediatric Unit of our Institution between May 2024 and October 2024. The inclusion criteria for both cases and controls were as follows: age 4 to 18 years; absence of comorbidities with an impact on body composition such as electrolyte disorders, extensive skin issues, nephrotic syndrome, heart and liver failure, dehydration, uncontrolled thyreopathy, uncontrolled celiac disease, hypocortisolism, severe obesity (defined as a BMI > 99° pct according to WHO tables for sex and age); absence of use of drugs that can impact body composition, such as systemic corticosteroids, hormonal therapies, fluid therapies, or previous chemotherapy.

Controls were prospectively recruited as the two next children attending the Pediatric Endocrinology Clinic of our Hospital with minor endocrine disorders such as thyroid nodules with normal function and isolated premature pubarche, non-severe familial short stature, and other minor conditions such as enuresis or innocent heart murmurs. Controls were matched for pubertal status using the Tanner scale, as it is among the main determinants of body composition during childhood and adolescence [11]. The case–control ratio was 1:2.

### Procedures and measurements

A wall-mounted stadiometer was used to measure body height to the nearest 0.1 cm in an upright position and barefoot. Body weight was assessed with a digital scale with an accuracy of 0.1 kg. Body mass index (BMI) was calculated as weight (kg)/height (m)^2^. BMI *z*-scores were calculated using PediTools Electronic Growth Chart Calculators based on Centres for Disease Control growth charts. The pubertal status was assessed using the Tanner scale.

In the T1D group, glucose profiles referred to the two weeks before BIA measurement were obtained from CGM or FGM. The reports included: time in range (TIR%), time above range (TAR%), and time below range (TBR%). Glycated hemoglobin (HbA1c%) was measured by DCA Vantage® Analyzer Siemens as part of the routine evaluation. Insulin delivery method (multiple daily injections—MDI; insulin pump: tubeless insulin pump or advanced hybrid closed loop—AHCL) was recorded, and total daily insulin dose was estimated on the basis of the average daily insulin requirement.

All participants were interviewed and asked whether they practiced any extracurricular physical activity; if so, they were classified in the “active lifestyle” group. No validated questionnaires were used to characterize physical activity.

BIA was performed using BIA-ACC® analyzer (Biotekna s.r.l., Marcon, Venice), a dual-frequency device that uses a high (50 kHz) and a low (1.5 kHz) frequency [12]. The measurements were carried out in a standard clinical setting during outpatient visits, and fasting was not required; participants did not practice any physical activity in the previous 2 h. Patients were in a supine position with legs slightly spread and arms not touching the body, on a non-electricity-conducting surface. Disposable electrodes (BioTekna) were used; for the upper extremities, electrodes were placed on the right hand, one on the metacarpal and one on the wrist areas, and for the lower extremities, electrodes were placed on the right foot, one on the metatarsal and one on the ankle areas.

The results were transferred and analyzed by specialized software (BioTekna Plus), and the specific parameters were assessed by the software using algorithms and validated with reference standards.

The BIA variables analyzed were fat mass percentage (FM%), fat-free mass percentage (FFM%), total body water percentage (TBW%), extracellular water percentage (ECW%), intracellular water percentage (ICW%), and basal metabolic rate (BMR, kg/day). Moreover, BIA allows also the estimation of phase angle degree (PA°) derived from conditions under 50 kHz according to the following formula: phase angle 50 = arctangent (reactance at 50 kHz/resistance at 50 kHz) [13]. Fat mass index (FMI) was calculated as fat mass (kg)/height (m)^2^.

### Statistical analysis

A formal sample size calculation could not be performed a priori due to the lack of preliminary information that would serve as a basis for the calculation. Thus, the study included a convenient sample with a 1:2 case:control ratio, where the cases were all eligible subjects (children and adolescents with T1D) who were referred to our unit during the study period. Data were summarized as frequency and percentage (categorical data) or median and interquartile range (numerical data). Comparisons between groups were performed using Mann–Whitney test, Kruskal–Wallis test, and Chi-Square test. In the comparison of BIA parameters between cases and controls, the effect sizes were reported as median difference with 95% confidence interval. Correlations between numerical variables were assessed using Spearman rank correlation coefficient. Subgroup analyses by age categories (4–10 years, 11–18 years) were also carried out given the broad age range spanning different developmental stages. Adjustment for multiple testing was not applied given the exploratory (rather than confirmatory) nature of the study. All tests were two-sided, and a *p*-value less than 0.05 was considered significant. Statistical analysis was performed using R 4.4 (R Foundation for Statistical Computing, Vienna, Austria) [14].

## Results

### Baseline participant characteristics

The analysis included 46 cases and 92 controls. Age and sex were comparable between patients and controls (Table 1).

**Table 1:** Baseline characteristics in patients with T1D and controls matched for Tanner stage

Variable	Patients with T1D (*n* = 46)	Controls (*n* = 92)	*P*-value
Tanner stage			
1	16 (35%)	32 (35%)	
2	2 (4%)	4 (4%)	
3	4 (9%)	8 (9%)	
4	7 (15%)	14 (15%)	
5	17 (37%)	34 (37%)	
Females	22 (48%)	49 (53%)	0.67
Age, years	13.7 (10.7; 15.1)	12.9 (9.7; 15.4)	0.77

*T1D* type 1 diabetes. Data summarized as *n* (%) or median (IQR). Tanner stage was not compared between cases and controls because they were matched for this variable during the participant selection

Cases and controls had comparable weight (*p* = 0.63), height (*p* = 0.97), BMI (*p* = 0.59), and BMI *z*-score (*p* = 0.74), as well as prevalence of active lifestyle (*p* = 0.19) (Table 2).

**Table 2:** Weight, height, BMI, BMI z-score, active lifestyle in patients with T1D and controls

Variable	Patients with T1D (*n* = 46)	Controls (*n* = 92)	*P*-value
Weight, kg	48.7 (33.8; 59.0)	49.8 (31.3; 65.2)	0.63
Height, cm	155.0 (139.5; 166.8)	153.5 (134.9; 166.2)	0.97
BMI, kg/m^2^	19.3 (17.4; 21.6)	19.4 (16.3; 23.8)	0.59
BMI *z*-score	0.2 (− 0.3; 0.8)	0.3 (− 0.3; 1.2)	0.74
Active lifestyle	21 (46%)	52/88 (59%)	0.19

*BMI* body mass index; *T1D* type 1 diabetes. Data summarized as *n* (%) or median (IQR)

Among patients with T1D, the median level of HbA1c% was 7.5 (IQR 6.9; 8.0), and the mean level of HbA1c% was 7.7 (standard deviation 1.2). The median activity time of the sensors used to monitor blood glucose levels was 97.2%. Data from glucose sensors showed a median TIR% of 61% (IQR 46.2; 71.0). Half of our patients used MDI (*n* = 23, 50%), whereas the other half used insulin pump (tubeless insulin pump *n* = 10, 22%; AHCL *n* = 13, 28%).

Further details about the cases are reported in Table 3.

**Table 3:** Information on patients with T1D

Variable	Patients with T1D (*n* = 46)
Disease duration, months	48.0 (14.5; 82.5)
HbA1c, %	7.5 (6.9; 8.0)
CGM/FGM activity time, %	97.2 (93.9; 98.6)
TIR last 2 weeks, %	61.0 (46.2; 71.0)
TAR last 2 weeks, %	32.0 (25.2; 48.7)
TBR last 2 weeks, %	2.0 (1.0; 4.4)
Total daily insulin dose, U/kg/die	0.6 (0.4; 0.7)
Insulin delivery method: MDI Tubeless insulin pump AHCL	23 (50%) 10 (22%) 13 (28%)

*AHCL* advanced hybrid closed loop; *CGM* continuous glucose monitoring; *FGM* flash glucose monitoring; *HbA1c* glycated hemoglobin; MDI, multiple daily injections; *TAR *time above range; *TBR* time below range; *TBW* total body water; *TIR* time in range; *T1D *type 1 diabetes. Data summarized as *n* (%) or median (IQR)

### BIA analysis results

The comparison of BIA parameters between cases and controls is summarized in Table 4. PA° was lower in patients with T1D than in controls (*p* = 0.02), while the other BIA data were not statistically different between cases and controls (Table 4). The comparison of BIA parameters between cases and controls within children aged 4–10 years and children aged 11–18 years is reported in Supplementary Tables 1 and 2. The sample size reduction prevented finding any statistically significant differences, but the confidence intervals may provide useful information for the reader.

**Table 4:** BIA parameters in cases and controls

BIA data	Patients with T1D (*n* = 46)	Controls (*n* = 92)	*P*-value	Median difference (95% confidence interval)
FM%	19.0 (14.2; 27.0)	19.0 (9.0; 31.0)	0.96	0.0 (− 5.0 to 4.5)
FFM%	81.0 (73.0; 85.7)	81.0 (69.0; 91.0)	0.96	0.0 (− 4.5 to 5.5)
FMI, kg/m^2^	3.8 (2.5; 5.7)	3.6 (1.4; 7.3)	0.96	0.2 (− 1.2 to 1.1)
TBW%	52.0 (44.2; 59.0)	51.5 (44.7; 60.2)	0.91	0.5 (− 5.5 to 5.0)
ECW%	46.0 (44.0; 49.0)	46.0 (42.0; 49.0)	0.43	0.0 (− 1.0 to 1.0)
ICW%	54.0 (51.0; 56.0)	54.0 (51.0; 58.0)	0.43	0.0 (− 1.0 to 1.0)
BMR, kcal/day	1200.5 (999.5; 1300.0)	1196.0 (966.0; 1357.0)	0.55	3.5 (− 116.0 to 98.0)
PA°	1.4 (1.0; 2.0)	1.9 (1.4; 2.3)	0.02	− 0.5 (− 0.7 to − 0.1)

*BIA* bioelectrical impedance analysis; *BMR* basal metabolic rate; *ECW* extracellular water; *FFM *fat free mass; *FM* fat mass; *FMI* fat mass index; *ICW* intracellular water; *PA° *phase angle; *TBW* total body water; *T1D *type 1 diabetes. Data summarized as median (IQR)

**Fig. 1:**
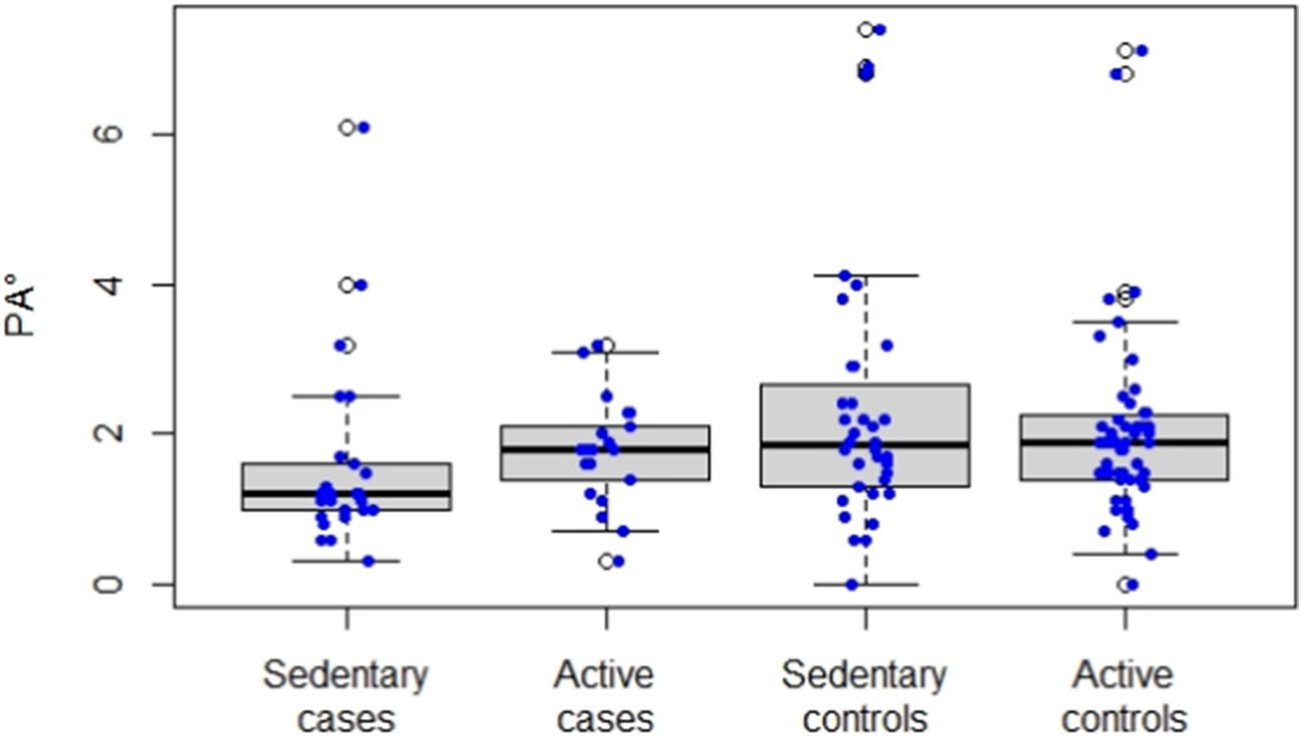
Phase angle (PA°) stratified by physical activity in cases and controls: box and whiskers plot

Median PA° was 1.2 (IQR 1.0–1.6) in sedentary cases, 1.8 (IQR 1.4–2.1) in active cases, 1.8 (IQR 1.3–2.5) in sedentary controls, and 1.9 (IQR 1.4–2.2) in active controls (*p* = 0.02, Fig. 1).

Among cases, an active lifestyle was associated with higher PA° (median 1.8; IQR 1.4–2.1) compared to sedentary habits (median 1.2; IQR 1.0–1.6) (*p* = 0.04), while no significant associations were found with the other BIA variables (Supplementary Table 3).

**Fig. 2:**
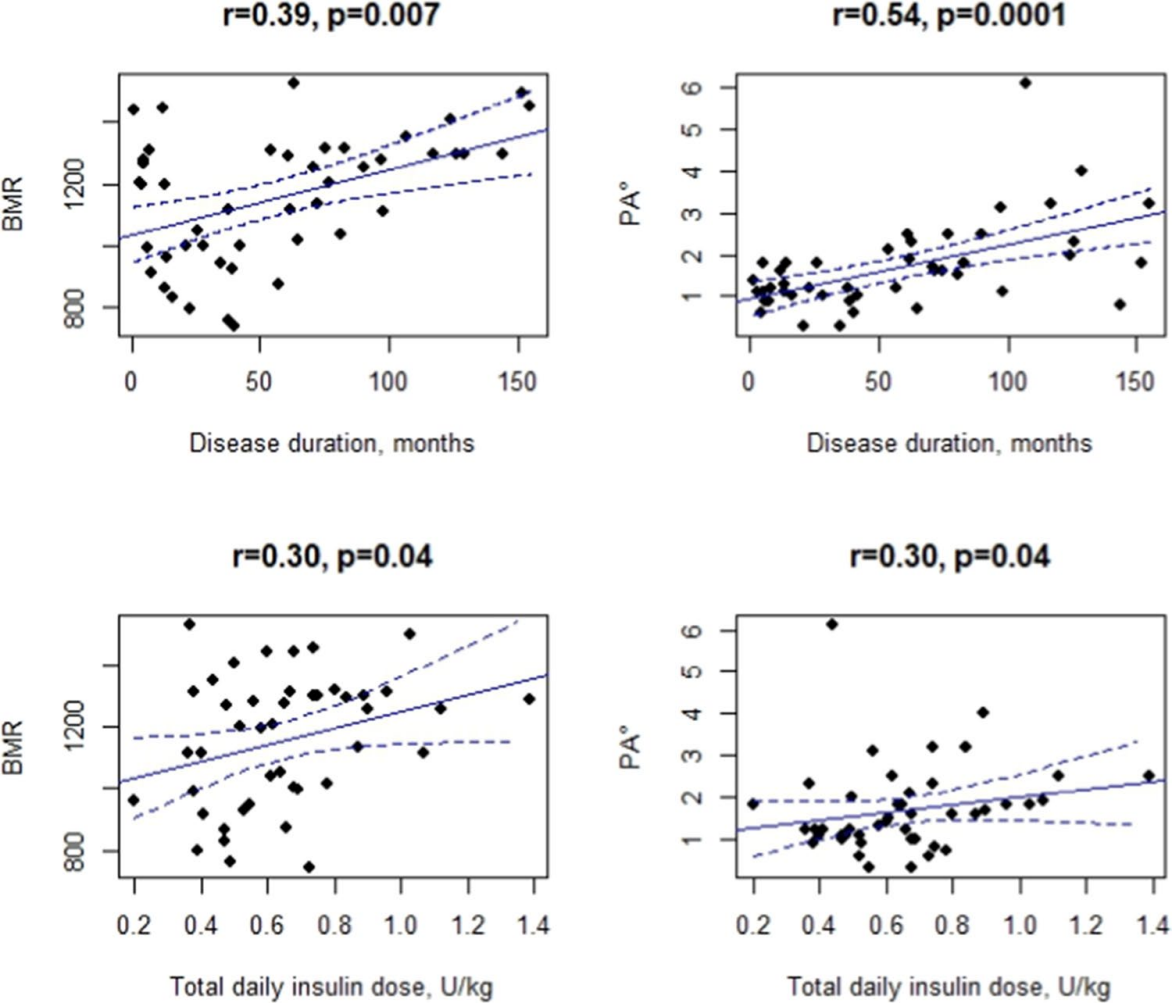
Correlation between BIA basal metabolic rate (BMR) and phase angle (PA°), and disease duration and total daily insulin dose among the cases: scatter plots with regression lines (solid lines) and confidence intervals (dashed lines)

Longer disease duration was correlated with higher BMR (*r* = 0.39, *p* = 0.007) and PA° (*r* = 0.54, *p* = 0.00019) (Fig. 2, Supplementary Table 4). Higher total daily insulin dose was correlated with higher BMR (*r* = 0.30, *p* = 0.04) and PA° (*r* = 0.30, *p* = 0.04) (Fig. 2, Supplementary Table 4).

The subgroup analyses by age categories suggested that the relationships between disease duration and BMR, between disease duration and PA°, and between total daily insulin dose and PA° may be different within children aged 4–10 years and children aged 11–18 years, but the limited sample size of the subgroups prevented drawing definitive conclusions (Supplementary Figs. 1–4).

Older age was correlated with longer disease duration (*r* = 0.58, *p* < 0.0001) and higher total daily insulin dose (*r* = 0.39, *p* = 0.007).

Insulin delivery method (MDI, tubeless insulin pump, or AHCL) was not associated with BIA variables (Supplementary Table 5).

## Discussion

The most important finding of the present study is the lower PA°, a BIA-derived parameter [8], in children with T1D compared to control subjects. PA° is an indicator of cellular performance, and its reduction suggests the occurrence of cellular stress in the diabetic population. By contrast, FM%, FFM%, FMI, TBW%, ICW%, and ECW% were comparable between patients and controls.

Beyond common body composition measurements, the present study employed BIA-ACC®, which is a dual-frequency bioelectrical impedance analysis device that can provide insights on cellular health by evaluating PA° and the energy expenditure through the assessment of basal metabolic rate (BMR). PA° is a direct measure of the electric properties of body tissues [15]. Oxidative stress and inflammation can cause cellular injuries, affecting cell structure and integrity and consequently causing an altered cellular water content distribution. Deranged fluid distribution can be efficiently detected by PA° variations, and as a consequence, PA° can be used as an early indicator of cellular health [16].

Assessment of PA° may represent a useful tool to identify patients that are at an increased risk of deranged body composition, as it was the only detectable BIA abnormality in our cohort of children with T1D and as it was better in those with an active lifestyle.

The hypothalamic–pituitary–adrenal (HPA) axis is related to cortisol release; a flattened or reversed circadian cortisol curve can be observed in patients exposed to chronic stress and inflammation, and derangement of the circadian cortisol curve can be associated with poor cellular function and altered fluid distribution. As PA° measures altered fluid distribution, studies suggest that it can be also considered an index of the HPA axis [13]. Therefore, we can speculate that in T1D, chronic low-grade inflammation appears early in the disease and is persistent over time [17, 18]. In addition, patients with diabetes are more exposed to psychological stress due to the demanding daily management of the disease [19, 20]. We hypothesized that our patients, despite showing acceptable glycemic control, still suffered from the effects of chronic inflammation and increased stress levels, potentially causing alterations in the HPA axis and contributing to reduced PA°.

Low PA° values have been already described in many pathological conditions in adults [21, 22] and children [23], and PA° has also been analyzed as a prognostic factor in a recent review suggesting an association between lower PA° and poor outcomes in hospitalized children [24].

Previous studies have already reported lower PA° in pediatric patients with T1D compared to peers at diabetes onset [9] and later on during the disease course [10]. In the same study, patients with diabetes showed higher FMI compared to controls, while this finding was not observed in our data. Of note, some relevant differences in terms of settings and patient characteristics should be underlined, as the previous study was conducted in Uganda (where there is a high burden of malnutrition and infectious diseases) and all patients were on insulin therapy using MDI or Mixtard without access to advanced devices to deliver insulin. All participants used sensors (CGM or FGM) with a high percentage of sensor usage time, ensuring the reliability of the data, and spent very low time in hypoglycemia and acceptable time in hyperglycemia [25]. Although the median TIR was 61%, not optimal according to the present recommendation for the pediatric population, our patients were characterized by generally satisfactory glycemic control consistent with the Italian pediatric diabetic population, therefore representative of a real-world population of pediatric patients with T1D [26].

It should be also be considered that the good TIR and the low TBR in our patients were also obtained thanks to the use of advanced devices to deliver insulin (tubeless insulin pumps or AHCL). Additionally, approximately half of our patients declared that they regularly practiced extracurricular sports.

PA° has been shown to be higher in individuals practicing regular sports [27, 28], and regular physical activity induces adaptation of the HPA axis to stress [29].

In our patients with diabetes, PA° significantly differed based on an active or non-active lifestyle, and patients with diabetes engaging in regular extracurricular physical activity showed PA° values comparable to those of the control group, probably due to better stress adaptation. The benefits of an active lifestyle on body composition in young patients affected by T1D have been recently confirmed in a study that showed lowering body fat in those practicing physical activity [30]. However, our data did not show further differences in other BIA parameters according to an active lifestyle, suggesting that PA° may be a more sensitive parameter when studying cellular well-being.

Our patients showed no significant differences in BMI or BMI z-score compared to controls, despite previous studies reporting an increased prevalence of overweight and obesity in children and adolescents with T1D [31–33].

BMR, the energy required to sustain vital functions [34], was comparable between the two groups, showing a balanced energy expenditure in patients with diabetes, unlike in cases of uncontrolled disease [35]. No other correlations were found between body composition and glycemic control parameters (HbA1c%, TIR%, TAR%, and TBR%) (Supplementary Table 4) or insulin delivery method (Supplementary Table 5).

Higher PA° and BMR were correlated, in the present study, to longer disease duration and higher daily insulin dose, but it should be noted that PA° physiologically increases with age in the first two decades of life, with a marked increment after puberty [36], and so does energy expenditure [37]. It is intuitive that disease duration is directly related to the age of the patients. Moreover, age and pubertal status are fundamental determinants of insulin requirements [38, 39]. Unfortunately, due to the strict correlation of older age to longer disease duration and higher total daily insulin dose in our sample, we were unable to determine if PA° and BMR increased with disease duration and insulin requirement as a sole consequence of the increased age. Higher PA° values could also be the consequence of better glycemic control reached after the start of insulin therapy, which is expected to improve as time passes from disease onset. Indeed, a previous study reported a decrease in PA° at T1D onset, followed by an improvement some months later [9].

Elevated BMR in people suffering from diabetes has been described, and some causes have been hypothesized, including impaired mitochondrial function and poor glycemic control with consequent glucosuria and gluconeogenesis [35, 40]. A higher daily insulin dose can be necessary due to increased food intake, and it can be responsible for an increase in BMR itself [41].

To our knowledge, this is the first study using BIA-ACC® to assess and compare body composition in children and adolescents with T1D and healthy peers. Moreover, our findings suggest that an active lifestyle can help children and adolescents with T1D increase PA° to level their healthy peers. The strengths of this study include the prospective design and the inclusion of cases and controls who were matched for pubertal status. However, our study had also some limitations that should be noted. The limited sample size did not allow us to fully explore if increasing age might have influenced the relationship between BMR, PA°, disease duration, and daily insulin dose. The analysis did not include any adjustments for multiple testing because of the exploratory purpose of the study; nonetheless, we acknowledge that multiple testing inflates type I error, hence our findings should be interpreted with caution and require confirmation in further investigations. No validated questionnaires were used to characterize physical activity. Moreover, the generalizability of the findings should be limited to similar subjects and settings. To our knowledge, there are no validation studies in the literature providing reference ranges for BIA-ACC in pediatric populations. However, the device can be also used in this population, with the same equations as adults for the percentage values analyzed in our study (FM%, FFM%, TBW%, ECW%, ICW%), for PA° and BMR. As a consequence, is not yet possible to determine if the differences found between cases and controls in our study are clinically meaningful, although plausible; the issue will be tested in future follow-up studies. Further studies may investigate the potential role of age in the relationship between BMR, PA°, disease duration, and daily insulin dose, and the impact of different physical activities on PA° levels.

## Conclusion

Children and adolescents with good management of T1D showed comparable body composition measurements but a lower phase angle to their healthy peers. Patients with T1D practicing extracurricular physical activity had PA° values comparable to healthy subjects, demonstrating the importance of an active lifestyle in those patients and suggesting impaired cellular function in those with sedentary habits. To implement the follow-up evaluation of children affected by T1D with BIA measures will allow detection of PA° abnormalities that will alert clinicians to possible body composition alterations at an early stage, allowing particular efforts both to ensure optimal glycaemic control and to encourage physical activity.

## Supplementary Information

Below is the link to the electronic supplementary material.

Supplementary Information 1 (DOCX 3.87 MB)

## Data Availability

Data is provided within the manuscript or Supplementary Information files.

## Abbreviations

AHCL: Advanced hybrid closed loop
BIA: Bioelectrical impedance analysis
BMI: Body mass index
BMR: Basal metabolic rate
CGM: Continuous glucose monitoring
ECW: Extracellular water
FGM: Flash glucose monitoring
FFM: Fat free mass
FM: Fat mass
FMI: Fat mass index
HbA1c: Glycated hemoglobin
HPA: Hypothalamic–pituitary–adrenal
ICW: Intracellular water
MDI: Multiple daily injections
PA°: Phase angle
TAR: Time above range
TBR: Time below range
TBW: Total body water
TIR: Time in range
T1D: Type 1 diabetes

## References

[1] Calella P, Gallè F, Fornelli G, Liguori G, Valerio G (2020) Type 1 diabetes and body composition in youth: a systematic review. Diabetes Metab Res Rev. 10.1002/dmrr.3211 31352688 10.1002/dmrr.3211

[2] Pollakova D, Tubili C, Di Folco U, De Giuseppe R, Battino M, Giampieri F (2023) Muscular involvement in long-term type 1 diabetes: does it represent an underestimated complication? Nutrition 112:112060. 10.1016/j.nut.2023.112060 37267657 10.1016/j.nut.2023.112060

[3] Zheng Y, Rostami Haji Abadi M, Gough J, Johnston JJD, Nour M, Kontulainen S (2022) Higher body fat in children and adolescents with type 1 diabetes–a systematic review and meta-analysis. Front Pediatr. 10.3389/fped.2022.911061 35813369 10.3389/fped.2022.911061 PMC9263393

[4] Silva AM, Campa F, Stagi S, Gobbo LA, Buffa R, Toselli S, Silva DAS, Gonçalves EM, Langer RD, Guerra-Júnior G, Machado DRL, Kondo E, Sagayama H, Omi N, Yamada Y, Yoshida T, Fukuda W, Gonzalez MC, Orlandi SP, Koury JC, Moro T, Paoli A, Kruger S, Schutte AE, Andreolli A, Earthman CP, Fuchs-Tarlovsky V, Irurtia A, Castizo-Olier J, Mascherini G, Petri C, Busert LK, Cortina-Borja M, Bailey J, Tausanovitch Z, Lelijveld N, Ghazzawi HA, Amawi AT, Tinsley G, Kangas ST, Salpéteur C, Vázquez-Vázquez A, Fewtrell M, Ceolin C, Sergi G, Ward LC, Heitmann BL, da Costa RF, Vicente-Rodriguez G, Cremasco MM, Moroni A, Shepherd J, Moon J, Knaan T, Müller MJ, Braun W, García-Almeida JM, Palmeira AL, Santos I, Larsen SC, Zhang X, Speakman JR, Plank LD, Swinburn BA, Ssensamba JT, Shiose K, Cyrino ES, Bosy-Westphal A, Heymsfield SB, Lukaski H, Sardinha LB, Wells JC, Marini E (2023) The bioelectrical impedance analysis (BIA) international database: aims, scope, and call for data. Eur J Clin Nutr 77:1143–1150. 10.1038/s41430-023-01310-x 37532867 10.1038/s41430-023-01310-x

[5] Ward LC, Brantlov S (2023) Bioimpedance basics and phase angle fundamentals. Rev Endocr Metab Disord 24:381–391. 10.1007/s11154-022-09780-3 36749540 10.1007/s11154-022-09780-3 PMC10140124

[6] Cvijetić S, Keser I, Boschiero D, Ilich JZ (2024) Prevalence of osteosarcopenic adiposity in apparently healthy adults and appraisal of age, sex, and ethnic differences. J Pers Med 14:782. 10.3390/jpm14080782 39201974 10.3390/jpm14080782 PMC11355127

[7] Houttu N, Kalliomäki M, Grönlund M-M, Niinikoski H, Nermes M, Laitinen K (2020) Body composition in children with chronic inflammatory diseases: a systematic review. Clin Nutr 39:2647–2662. 10.1016/j.clnu.2019.12.027 32035751 10.1016/j.clnu.2019.12.027

[8] Kumar S, Dutt A, Hemraj S, Bhat S, Manipadybhima B (2012) Phase angle measurement in healthy human subjects through bio-impedance analysis. Iran J Basic Med Sci 15:1180–1184 23653848 PMC3646229

[9] Więch P, Bazaliński D, Sałacińska I, Binkowska-Bury M, Korczowski B, Mazur A, Kózka M, Dąbrowski M (2018) Decreased bioelectrical impedance phase angle in hospitalized children and adolescents with newly diagnosed type 1 diabetes: a case-control study. J Clin Med 7:516. 10.3390/jcm7120516 30518100 10.3390/jcm7120516 PMC6306918

[10] Nsamba J, Eroju P, Drenos F, Mathews E (2022) Body composition characteristics of type 1 diabetes children and adolescents: a hospital-based case-control study in Uganda. Children (Basel) 9:1720. 10.3390/children9111720 36360448 10.3390/children9111720 PMC9688493

[11] Siervogel RM, Demerath EW, Schubert C, Remsberg KE, Chumlea WC, Sun S, Czerwinski SA, Towne B (2003) Puberty and body composition. Horm Res Paediatr 60:36–45. 10.1159/000071224 10.1159/000071224 12955016

[12] Chrousos GP, Papadopoulou-Marketou N, Bacopoulou F, Lucafò M, Gallotta A, Boschiero D (2022) Photoplethysmography (PPG)-determined heart rate variability (HRV) and extracellular water (ECW) in the evaluation of chronic stress and inflammation. Hormones (Athens) 21:383–390. 10.1007/s42000-021-00341-y 35028916 10.1007/s42000-021-00341-y

[13] Tsigos C, Stefanaki C, Lambrou GI, Boschiero D, Chrousos GP (2015) Stress and inflammatory biomarkers and symptoms are associated with bioimpedance measures. Eur J Clin Invest 45:126–134. 10.1111/eci.12388 25431352 10.1111/eci.12388

[14] R Core Team (2024) R: a language and environment for statistical computing. R Foundation for Statistical Computing, Vienna, Austria. https://www.r-project.org/. Accessed 10 Oct 2024

[15] Lukaski HC, Garcia-Almeida JM (2023) Phase angle in applications of bioimpedance in health and disease. Rev Endocr Metab Disord 24:367–370 36944817 10.1007/s11154-023-09799-0 PMC10030341

[16] da Silva BR, Orsso CE, Gonzalez MC, Sicchieri JMF, Mialich MS, Jordao AA, Prado CM (2023) Phase angle and cellular health: inflammation and oxidative damage. Rev Endocr Metab Disord 24(3):543–562. 10.1007/s11154-022-09775-0 36474107 10.1007/s11154-022-09775-0 PMC9735064

[17] Devaraj S, Glaser N, Griffen S, Wang-Polagruto J, Miguelino E, Jialal I (2006) Increased monocytic activity and biomarkers of inflammation in patients with type 1 diabetes. Diabetes 55:774–779. 10.2337/diabetes.55.03.06.db05-1417 16505242 10.2337/diabetes.55.03.06.db05-1417

[18] Simeunovic A, Brunborg C, Heier M, Seljeflot I, Dahl-Jørgensen K, Margeirsdottir HD (2023) Sustained low-grade inflammation in young participants with childhood onset type 1 diabetes: the Norwegian atherosclerosis and childhood diabetes (ACD) study. Atherosclerosis 379:117151. 10.1016/j.atherosclerosis.2023.05.020 37349194 10.1016/j.atherosclerosis.2023.05.020

[19] Ingrosso DMF, Primavera M, Samvelyan S, Tagi VM, Chiarelli F (2023) Stress and diabetes mellitus: pathogenetic mechanisms and clinical outcome. Horm Res Paediatr 96:34–43 35124671 10.1159/000522431

[20] Rechenberg K, Whittemore R, Holland M, Grey M (2017) General and diabetes-specific stress in adolescents with type 1 diabetes. Diabetes Res Clin Pract 130:1–8. 10.1016/j.diabres.2017.05.003 28551480 10.1016/j.diabres.2017.05.003 PMC5608607

[21] Murakami T, Kobayashi T, Ono H, Shibuma H, Tsuji K, Nikkuni E, Mori N, Ohkouchi S, Tabata M, Irokawa T, Ogawa H, Takahashi T, Kurosawa H (2024) Phase angle as an indicator of sarcopenia and malnutrition in patients with chronic obstructive pulmonary disease. Respir Investig 62:651–656. 10.1016/j.resinv.2024.05.012 38761479 10.1016/j.resinv.2024.05.012

[22] Ruiz-Margáin A, Macías-Rodríguez RU, Ampuero J, Cubero FJ, Chi-Cervera L, Ríos-Torres SL, Duarte-Rojo A, Espinosa-Cuevas Á, Romero-Gómez M, Torre A (2016) Low phase angle is associated with the development of hepatic encephalopathy in patients with cirrhosis. World J Gastroenterol 22:10064. 10.3748/wjg.v22.i45.10064 28018114 10.3748/wjg.v22.i45.10064 PMC5143753

[23] Więch P, Dabrowski M, Bazaliński D, Sałacińska I, Korczowski B, Binkowska-Bury M (2018) Bioelectrical impedance phase angle as an indicator of malnutrition in hospitalized children with diagnosed inflammatory bowel diseases—a case control study. Nutrients 10. 10.3390/nu10040499 10.3390/nu10040499 PMC5946284 29673210

[24] Fernández-Jiménez R, Martín-Masot R, Cornejo-Pareja I, Vegas-Aguilar IM, Herrador-López M, Tinahones FJ, Navas-López VM, Bellido-Guerrero D, García-Almeida JM (2023) Phase angle as a marker of outcome in hospitalized pediatric patients. A systematic review of the evidence (GRADE) with meta-analysis. Rev Endocr Metab Disord 24:751–765. 10.1007/s11154-023-09817-1 37486555 10.1007/s11154-023-09817-1 PMC10404571

[25] Urakami T (2022) Significance of the CGM metric of time in range in children and adolescents with type 1 diabetes. Endocr J 69:EJ22-0257. 10.1507/endocrj.EJ22-0257 10.1507/endocrj.EJ22-0257 36002301

[26] Nicolucci A, Graziano G, Lombardo F, Rabbone I, Rossi MC, Vespasiani G, Zucchini S, Bonfanti R, Bracciolini GP, Cherubini V, Bobbio A, Zucchini S, Suprani T, Donno V, Lombardo F, Bonfanti R, Franzese A, Rabbone I, Graziani V, Zampolli M, Rutigliano I, deSanctis L, Guerraggio LP, Franceschi R, Tornese G, ranco FF, Maffeis C, Arnaldi C (2024) Continuous improvement of quality of care in pediatric diabetes: the ISPED CARD clinical registry. Acta Diabetol 61:599–607. 10.1007/s00592-023-02233-6 38332378 10.1007/s00592-023-02233-6 PMC11055792

[27] Mundstock E, Amaral MA, Baptista RR, Sarria EE, dos Santos RRG, Filho AD, Rodrigues CAS, Forte GC, Castro L, Padoin AV, Stein R, Perez LM, Ziegelmann PK, Mattiello R (2019) Association between phase angle from bioelectrical impedance analysis and level of physical activity: systematic review and meta-analysis. Clin Nutr 38:1504–1510. 10.1016/j.clnu.2018.08.031 30224304 10.1016/j.clnu.2018.08.031

[28] Yamada Y, Yoshida T, Murakami H, Kawakami R, Gando Y, Ohno H, Tanisawa K, Konishi K, Julien T, Kondo E, Nakagata T, Nanri H, Miyachi M (2022) Phase angle obtained via bioelectrical impedance analysis and objectively measured physical activity or exercise habits. Sci Rep 12:17274. 10.1038/s41598-022-21095-6 36241873 10.1038/s41598-022-21095-6 PMC9568532

[29] Athanasiou N, Bogdanis GC, Mastorakos G (2023) Endocrine responses of the stress system to different types of exercise. Rev Endocr Metab Disord 24:251–266 36242699 10.1007/s11154-022-09758-1 PMC10023776

[30] Rangel MA, Calejo R, Lopes V, Campos RA, Leite AL (2024) Body composition in a pediatric population with type-1 diabetes – the importance of planned physical exercise. Ann Endocrinol (Paris). 10.1016/j.ando.2024.09.004 39307238 10.1016/j.ando.2024.09.004

[31] Liu LL, Lawrence JM, Davis C, Liese AD, Pettitt DJ, Pihoker C, Dabelea D, Hamman R, Waitzfelder B, Kahn HS (2010) Prevalence of overweight and obesity in youth with diabetes in USA: the search for diabetes in youth study. Pediatr Diabetes 11:4–11. 10.1111/j.1399-5448.2009.00519.x 19473302 10.1111/j.1399-5448.2009.00519.x

[32] Maffeis C, Birkebaek NH, Konstantinova M, Schwandt A, Vazeou A, Casteels K, Jali S, Limbert C, Pundziute-Lycka A, Toth-Heyn P, de Beaufort C, Sumnik Z, Cherubini V, Svensson J, Pacaud D, Kanaka-Gantenbein C, Shalitin S, Bratina N, Hanas R, Alonso GT, Poran L, Pereira AL, Marigliano M (2018) Prevalence of underweight, overweight, and obesity in children and adolescents with type 1 diabetes: data from the international SWEET registry. Pediatr Diabetes 19:1211–1220. 10.1111/pedi.12730 30033651 10.1111/pedi.12730

[33] Minges KE, Whittemore R, Weinzimer SA, Irwin ML, Redeker NS, Grey M (2017) Correlates of overweight and obesity in 5529 adolescents with type 1 diabetes: the T1D exchange clinic registry. Diabetes Res Clin Pract 126:68–78. 10.1016/j.diabres.2017.01.012 28214669 10.1016/j.diabres.2017.01.012 PMC5401652

[34] Wideman TH, Sullivan MJL, Inada S, McIntyre D, Kumagai M, Yahagi N, Turner JR, Upton J, Burns RJ, Rothman AJ, Michie S, Johnston M, Nakashima M, Vedhara K, Dawe K, Wong C, Gellman MD, Brimmer D, Zielinski-Gutierrez E, Troxel W, Drerup M, Nakashima M, Barrett C, Gafni A, Birch S, Riley K, Turner JR, Gebel K, Ding D, Hooker SA, Hidalgo B, Meneghini L, Turner JR, Borsari B, Hustad J, Sherry S, Fitzpatrick S, Benedict C, Hashizume M, Shetty V, Miles E, Baumann LC, Karel A, France C, France JL, Carrillo A, Gomez-Meade C, Ginty AT, Hughes BM, Ginty AT, Ginty AT, Messiah S, Di Katie SM, Millstein R, Messiah S, Messiah S, Spiers M, Gidron Y, Haney A, Okun ML, Beaton EA, Beaton EA, Roy E, Harms V, Elias L, Hamann H, Stanton AL, Yanez BR, Masters KS, Drobnjak S, Baumann LC, Karel A, LaCaille L, Ding D, Patino-Fernandez AM, Kotlyar M, Vuchetich JP, Woltz P (2013) Basal metabolic rate. Encyclopedia of Behavioral Medicine. Springer, New York, New York, NY, pp 176–177

[35] Caron N, Peyrot N, Caderby T, Verkindt C, Dalleau G (2016) Energy expenditure in people with diabetes mellitus: a review. Front Nutr 3 10.3389/fnut.2016.00056 PMC5177618 28066773

[36] Ballarin G, Valerio G, Alicante P, Di Vincenzo O, Scalfi L (2022) Bioelectrical impedance analysis (BIA)-derived phase angle in children and adolescents: a systematic review. J Pediatr Gastroenterol Nutr 75:120–130. 10.1097/MPG.0000000000003488 35653386 10.1097/MPG.0000000000003488

[37] Pontzer H, Yamada Y, Sagayama H, Ainslie PN, Andersen LF, Anderson LJ, Arab L, Baddou I, Bedu-Addo K, Blaak EE, Blanc S, Bonomi AG, Bouten CVC, Bovet P, Buchowski MS, Butte NF, Camps SG, Close GL, Cooper JA, Cooper R, Das SK, Dugas LR, Ekelund U, Entringer S, Forrester T, Fudge BW, Goris AH, Gurven M, Hambly C, El Hamdouchi A, Hoos MB, Hu S, Joonas N, Joosen AM, Katzmarzyk P, Kempen KP, Kimura M, Kraus WE, Kushner RF, Lambert EV, Leonard WR, Lessan N, Martin C, Medin AC, Meijer EP, Morehen JC, Morton JP, Neuhouser ML, Nicklas TA, Ojiambo RM, Pietiläinen KH, Pitsiladis YP, Plange-Rhule J, Plasqui G, Prentice RL, Rabinovich RA, Racette SB, Raichlen DA, Ravussin E, Reynolds RM, Roberts SB, Schuit AJ, Sjödin AM, Stice E, Urlacher SS, Valenti G, Van Etten LM, Van Mil EA, Wells JCK, Wilson G, Wood BM, Yanovski J, Yoshida T, Zhang X, Murphy-Alford AJ, Loechl C, Luke AH, Rood J, Schoeller DA, Westerterp KR, Wong WW, Speakman JR (2021) Daily energy expenditure through the human life course. Science 373(1979):808–812. 10.1126/science.abe5017 34385400 10.1126/science.abe5017 PMC8370708

[38] Mitsui Y, Kuroda A, Ishizu M, Mori H, Kurahashi K, Kondo T, Yoshida S, Akehi Y, Aihara K-I, Endo I, Abe M, Matsuhisa M (2022) Basal insulin requirement in patients with type 1 diabetes depends on the age and body mass index. J Diabetes Investig 13:292–298. 10.1111/jdi.13547 33740836 10.1111/jdi.13547 PMC8847154

[39] Wiegand S, Raile K, Reinehr T, Hofer S, Näke A, Rabl W, Holl RW, __ (2008) Daily insulin requirement of children and adolescents with type 1 diabetes: effect of age, gender, body mass index and mode of therapy. Eur J Endocrinol 158:543–549. 10.1530/EJE-07-0904 10.1530/EJE-07-0904 18362302

[40] Monaco CMF, Perry CGR, Hawke TJ (2020) Alterations in mitochondrial functions and morphology in muscle and non-muscle tissues in type 1 diabetes: implications for metabolic health. Exp Physiol 105:565–570. 10.1113/EP088096 31826331 10.1113/EP088096

[41] Molé PA (1990) Impact of energy intake and exercise on resting metabolic rate. Sports Med 10:72–87. 10.2165/00007256-199010020-00002 2204100 10.2165/00007256-199010020-00002

