# Supplementary material for: Bioelectrical impedance analysis of body composition in children and adolescents with type 1 diabetes: a prospective case–control study
Source: Eur J Pediatr. 2025 Aug 18;184(9):560. doi: 10.1007/s00431-025-06401-4 (PMC12361262; doi:10.1007/s00431-025-06401-4)
Supplement: Supplementary file 1 — Supplementary Information 1 (DOCX 3.87 MB) [file 431_2025_6401_MOESM1_ESM.docx]

**BIOELECTRICAL IMPEDANCE ANALYSIS OF BODY COMPOSITION IN CHILDREN AND ADOLESCENTS WITH TYPE 1 DIABETES: A PROSPECTIVE CASE-CONTROL STUDY.**

Giardinelli S^1^, Lambertini AG^2^, Lumaca A^2^, Boschiero D^3^, Cavallin F^4^, Zucchini S^2^, Malaventura C^5*^, Suppiej A^5^

1. School of Pediatrics, Department of Medical Sciences, University of Ferrara, Ferrara, Italy
2. Diabetes and Endocrinology Clinic, Pediatric Unit, University Hospital of Ferrara, Ferrara Italy
3. BioTekna®, 30020 Marcon-Venice, Italy; [dario.boschiero@biotekna.com](mailto:dario.boschiero@biotekna.com).
4. Independent Statistician, 36020 Solagna, Italy
5. Department of Medical Sciences, Pediatric Section, University of Ferrara, Ferrara, Italy

*Corresponding Author: Malaventura C, Azienda Ospedaliera Universitaria Sant'Anna, Via Aldo Moro 8 - 44124, Ferrara, Italy; [mlvcst@unife.it](mailto:mlvcst@unife.it).

**Supplementary Table 1**. BIA parameters in cases and controls aged 4-10 years

| BIA data | Patients with T1D aged 4-10 years (n=14) | Controls aged 4-10 years (n=29) | p-value | Median difference (95% confidence interval) |
| --- | --- | --- | --- | --- |
| FM% | 13.0 (6.5; 18.8) | 7.0 (4.0; 17.0) | 0.18 | 6.0 (-1.5 to 11.0) |
| FFM% | 87.0 (81.2; 93.5) | 93.0 (83.0; 96.0) | 0.18 | -6.0 (-11.5 to 1.0) |
| FMI, kg/m^2^ | 2.1 (0.9; 3.6) | 1.2 (0.6; 3.1) | 0.22 | 0.9 (-0.3 to 2.3) |
| TBW% | 60.0 (47.5; 70.0) | 62.0 (55.0; 70.0) | 0.57 | -2.0 (-13.0 to 6.0) |
| ECW% | 48.5 (46.0; 50.7) | 50.0 (47.0; 55.0) | 0.19 | -1.5 (-7.0 to 1.0) |
| ICW% | 51.5 (49.2; 54.0) | 50.0 (45.0; 53.0) | 0.19 | 1.5 (-1.0 to 7.0) |
| BMR, kcal/day | 935.5 (838.8; 997.2) | 905.0 (837.0; 967.0) | 0.66 | 30.5 (-46.5 to 126.0) |
| PA° | 1.1 (0.9; 1.2) | 1.4 (0.8; 1.9) | 0.16 | -0.3 (-0.6 to 0.0) |

Data summarized as median (IQR). BIA, Bioelectrical Impedance Analysis; BMR, Basal Metabolic Rate; ECW, Extracellular Water; FFM, Fat Free Mass; FM, Fat Mass; FMI, Fat Mass Index; ICW, Intracellular Water; PA°, Phase Angle; TBW, Total Body Water; T1D, Type 1 Diabetes.

**Supplementary Table 2**. BIA parameters in cases and controls aged 11-18 years

| BIA data | Patients with T1D aged 11-18 years (n=32) | Controls aged 11-18 years (n=63) | p-value | Median difference (95% confidence interval) |
| --- | --- | --- | --- | --- |
| FM% | 22.0 (15.7; 27.2) | 26.0 (16.0; 32.5) | 0.25 | -4.0 (-8.5 to 2.0) |
| FFM% | 78.0 (72.7; 84.2) | 74.0 (67.5; 84.0) | 0.25 | 4.0 (-2.0 to 9.0) |
| FMI, kg/m^2^ | 4.4 (2.9; 6.0) | 5.4 (2.8; 8.59 | 0.30 | -1.0 (-2.7 to 0.5) |
| TBW% | 49.0 (43.7; 55.2) | 47.0 (43.0; 55.) | 0.48 | 2.0 (-1.5 to 7.0) |
| ECW% | 45.0 (42.7; 47.0) | 44.0 (41.0; 46.0) | 0.08 | 1.0 (-1.0 to 3.5) |
| ICW% | 55.0 (53.0; 57.2) | 56.0 (54.0; 59.0) | 0.08 | -1.0 (-3.0 to 1.0) |
| BMR, kcal/day | 1284.0 (1130.0; 1316.0) | 1314.0 (1184.0; 1391.0) | 0.15 | -30.0 (-97.0; 24.0) |
| PA° | 1.7 (1.2; 2.3) | 2.0 (1.5; 2.6) | 0.06 | -0.3 (-0.6 to 0.1) |

Data summarized as median (IQR). BIA, Bioelectrical Impedance Analysis; BMR, Basal Metabolic Rate; ECW, Extracellular Water; FFM, Fat Free Mass; FM, Fat Mass; FMI, Fat Mass Index; ICW, Intracellular Water; PA°, Phase Angle; TBW, Total Body Water; T1D, Type 1 Diabetes.

**Supplementary Table 3**. Association between BIA parameters and lifestyle in the cases

| BIA data | Sedentary Cases | Cases with active lifestyle | p-value |
| --- | --- | --- | --- |
| FM% | 17 (11; 26) | 23 (16; 27) | 0.14 |
| FFM% | 83 (74; 89) | 77 (73; 84) | 0.14 |
| FMI, kg/m^2^ | 3 (1.8; 4.6) | 4.6 (2.9; 5.7) | 0.11 |
| TBW% | 54 (46; 63) | 50 (44; 55) | 0.16 |
| ECW% | 46 (45; 49) | 46 (43; 49) | 0.50 |
| ICW% | 54 (51; 55) | 54 (51; 57) | 0.50 |
| BMR, kcal/day | 1196 (944; 1289) | 1277 (1050; 1406) | 0.05 |
| PA° | 1.2 (1.0; 1.6) | 1.8 (1.4; 2.1) | 0.04 |

Data summarized as median (IQR). BIA, Bioelectrical Impedance Analysis; BMR, Basal Metabolic Rate; ECW, Extracellular Water; FFM, Fat Free Mass; FM, Fat Mass; FMI, Fat Mass Index; ICW, Intracellular Water; PA°, Phase Angle; TBW, Total Body Water; T1D, Type 1 Diabetes.

**Supplementary Table 4**. Correlation between BIA information and relevant clinical characteristics (numerical variables) among the cases

| BIA data | BMI | BMI z-score | Disease duration, months | Total daily insulin dose, U/kg/die | HbA1c, % | TIR last 2 weeks, % | TAR last 2 weeks, % | TBR last 2 weeks, % |
| --- | --- | --- | --- | --- | --- | --- | --- | --- |
| FM% | 0.91 (p<0.0001) | 0.66 (p<0.0001) | -0.17 (p=0.26) | 0.09 (p=0.56) | -0.20 (p=0.19) | 0.17 (p=0.25) | -0.10 (p=0.52) | -0.16 (p=0.28) |
| FFM% | -0.91 (p<0.0001) | -0.66 (p<0.0001) | 0.17 (p=0.26) | -0.09 (p=0.56) | 0.20 (p=0.19) | -0.17 (p=0.25) | 0.10 (p=0.52) | 0.16 (p=0.28) |
| FMI, kg/m^2^ | 0.95 (p<0.0001) | 0.68 (p<0.0001) | 0.22 (p=0.14) | 0.09 (p=0.54) | -0.20 (p=0.19) | 0.14 (p=0.34) | -0.06 (p=0.69) | -0.20 (p=0.18) |
| TBW% | -0.64 (p<0.0001) | -0.32 (p=0.03) | -0.18 (p=0.23) | -0.17 (p=0.26) | 0.06 (p=0.70) | -0.10 (p=0.50) | 0.05 (p=0.73) | 0.05 (p=0.74) |
| ECW% | -0.63 (p<0.0001) | -0.40 (p=0.006) | -0.15 (p=0.33) | -0.02 (p=0.89) | 0.06 (p=0.71) | -0.03 (p=0.83) | 0.02 (p=0.90) | 0.19 (p=0.20) |
| ICW% | 0.63 (p<0.0001) | 0.40 (p=0.006) | 0.15 (p=0.33) | 0.02 (p=0.89) | -0.06 (p=0.71) | 0.03 (p=0.83) | -0.02 (p=0.90) | -0.19 (p=0.20) |
| BMR, kcal/day | 0.73 (p<0.0001) | 0.25 (p=0.09) | 0.39 (p=0.007) | 0.30 (p=0.04) | 0.03 (p=0.82) | -0.03 (p=0.83) | 0.05 (p=0.75) | -0.13 (p=0.38) |
| PA° | 0.63 (p<0.0001) | 0.29 (p=0.05) | 0.54 (p=0.0001) | 0.30 (p=0.04) | 0.15 (p=0.33) | -0.20 (p=0.17) | 0.25 (p=0.10) | -0.09 (p=0.55) |

Data reported as Spearman rank correlation coefficient with p-value within brackets. BIA, Bioelectrical Impedance Analysis; BMI, Body Mass Index; BMR, Basal Metabolic Rate; ECW, Extracellular Water; FFM, Fat Free Mass; FM, Fat Mass; FMI, Fat Mass Index; HbA1c, Glycated Hemoglobin; ICW, Intracellular Water; PA°, Phase Angle; TAR, Time Above Range; TBR, Time Below Range; TBW, Total Body Water; TIR, Time In Range; T1D, Type 1 Diabetes.

Supplementary Figure 1. Correlation between BIA BMR and disease duration among the cases aged 4-10 years and cases aged 11-18 years: scatter plots with regression lines (solid lines) and confidence intervals (dashed lines).


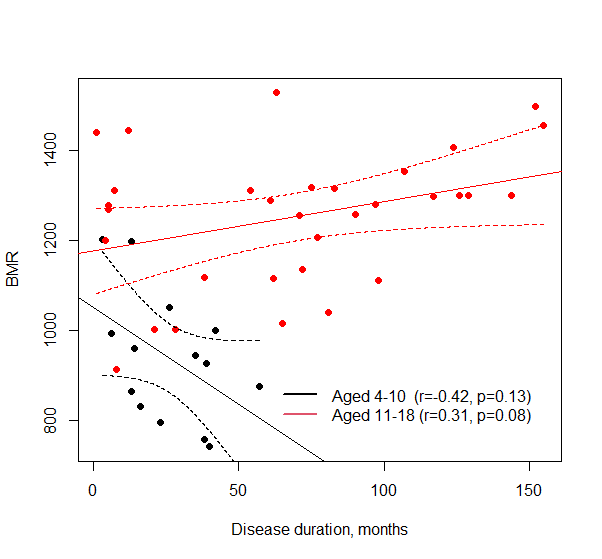


Supplementary Figure 2. Correlation between BIA PA° and disease duration among the cases aged 4-10 years and cases aged 11-18 years: scatter plots with regression lines (solid lines) and confidence intervals (dashed lines).


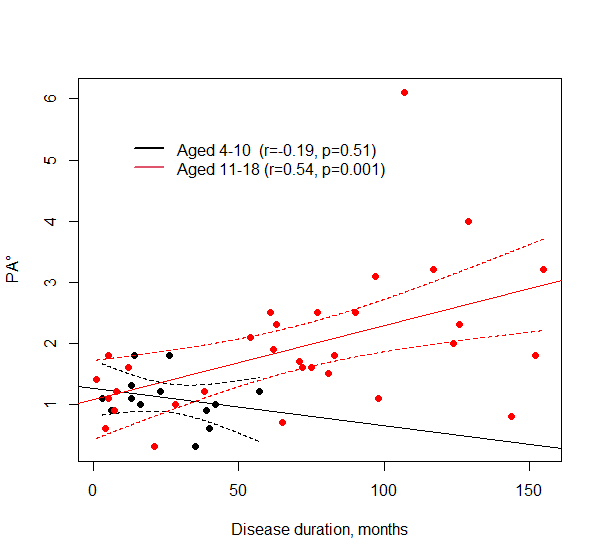


Supplementary Figure 3. Correlation between BIA BMR and total daily insulin dose among the cases aged 4-10 years and cases aged 11-18 years: scatter plots with regression lines (solid lines) and confidence intervals (dashed lines).


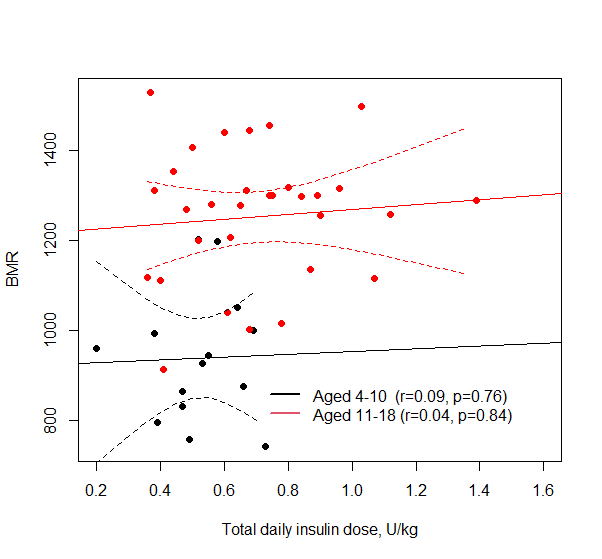


Supplementary Figure 4. Correlation between BIA PA° and total daily insulin dose among the cases aged 4-10 years and cases aged 11-18 years: scatter plots with regression lines (solid lines) and confidence intervals (dashed lines).


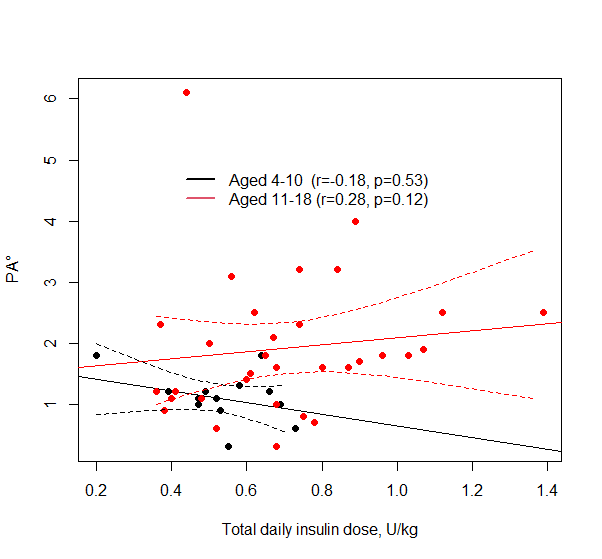


**Supplementary Table 5**. Association between BIA information and insulin delivery method in the cases

| BIA data | MDI | Tubeless insulin pump | AHCL | p-value |
| --- | --- | --- | --- | --- |
| FM% | 19 (12.5; 24) | 26.5 (15.8; 27.8) | 17 (15; 27) | 0.45 |
| FFM% | 81 (76; 87.5) | 73.5 (72.2; 84.2) | 83 (73; 85) | 0.45 |
| FMI, kg/m^2^ | 3.8 (2.1; 4.9) | 5.6 (2.9; 5.8) | 3.7 (2.6; 6.3) | 0.47 |
| TBW% | 54 (45.5; 59) | 46 (43.2; 51.2) | 55 (46; 62) | 0.22 |
| ECW% | 46 (44; 47.5) | 46 (46; 49) | 46 (44; 50) | 0.72 |
| ICW% | 54 (52.5; 56) | 54 (51; 54) | 54 (50; 56) | 0.72 |
| BMR, kcal/day | 1201 (1033; 1305) | 1230.5 (1112; 1294.8) | 1001 (944; 1296) | 0.51 |
| PA° | 1.3 (1; 1.8) | 2 (1.2; 2.4) | 1.5 (0.9; 2.3) | 0.36 |

Data summarized as median (IQR). AHCL, Advanced Hybrid Closed Loop; BIA, Bioelectrical Impedance Analysis; BMR, Basal Metabolic Rate; ECW, Extracellular Water; FFM, Fat Free Mass; FM, Fat Mass; FMI, Fat Mass Index; ICW, Intracellular Water; MDI, Multiple Daily Injections; PA°, Phase Angle; TBW, Total Body Water; T1D, Type 1 Diabetes.
